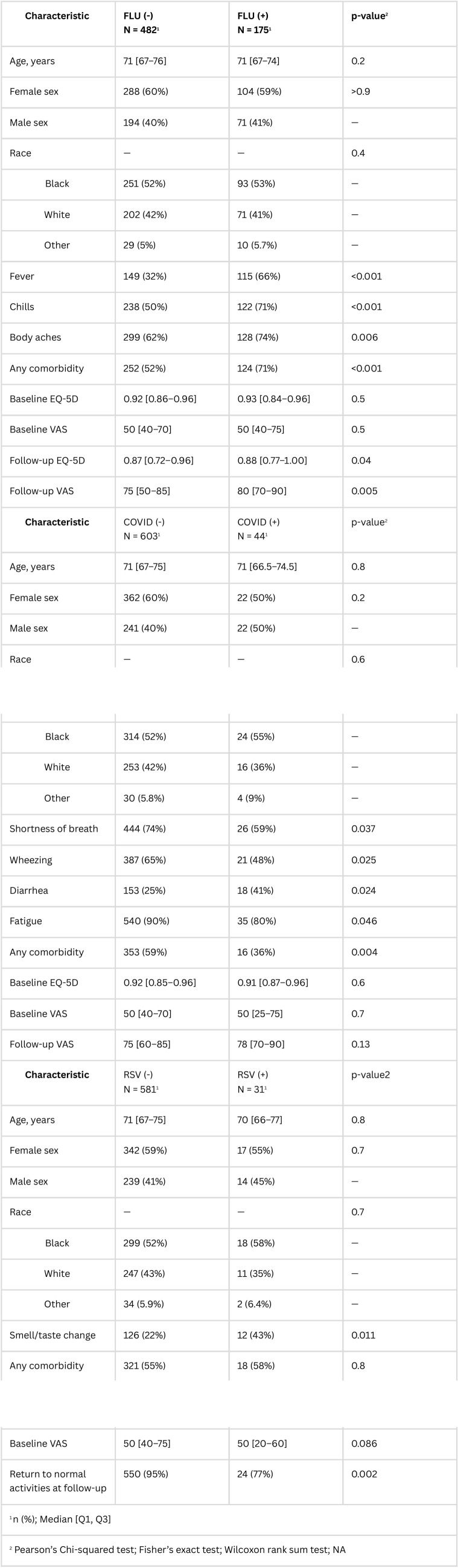# 371 Predictors of Hospital Onset Clostridioides Difficile Infection in a Kansas City Hospital

**DOI:** 10.1017/ash.2026.10707

**Published:** 2026-06-23

**Authors:** Luke Kabbara, Zainab Albar, Joy Abou Farah, Alexia El Khoury, Christopher Ladikos, Jay Krishnan, Elie Saade, University Hospitals Cleveland Medical Center

**Affiliations:** 1 Case Western Reserve University/ UH Hospitals; 2 Case Western Reserve University; 3 University Hospitals, Case Western Reserve University

## Abstract

**Background:** Respiratory viral infections cause substantial acute morbidity among older adults; however, their impact on short-term health-related quality of life (HRQoL) and functional recovery remains incompletely characterized. **Methods:** We conducted a prospective observational analysis among adults aged ?65 years enrolled in the U.S. Flu Vaccine Effectiveness Network. Multivariable linear regression was used to examine associations between respiratory virus test positivity (influenza, COVID-19, and respiratory syncytial virus [RSV]) and follow-up HRQoL. Outcomes included EQ-5D utility scores and self-rated health measured using a visual analog scale (VAS). Models adjusted for baseline HRQoL, demographics, household characteristics, and comorbidities. Bias-reduced CR2 cluster-robust standard errors were used to account for heteroskedasticity and within-site clustering. Multicollinearity was assessed using variance inflation factors, all of which were <2. EQ-5D coefficients were rescaled to a 0–100 metric to improve interpretability. **Results:** After multivariable adjustment, respiratory virus test positivity was not consistently associated with worse follow-up HRQoL. Influenza test positivity was not associated with follow-up EQ-5D scores (? = ?0.4 points on a 100-point EQ-5D scale, 95% CI ?12.5 to 11.6) and showed a positive but non-significant association with follow-up VAS scores (? = 5.9, 95% CI ?2.0 to 13.8). COVID-19 test positivity was associated with higher follow-up EQ-5D scores (? = 21.6, 95% CI 6.0–37.2), but not with follow-up VAS scores. RSV test positivity was not associated with follow-up EQ-5D (? = ?2.1, 95% CI ?16.6 to 12.4) or VAS scores (? = 13.6, 95% CI ?23.4 to 50.6). Across all EQ-5D models, baseline EQ-5D was the strongest predictor of follow-up EQ-5D. In contrast, baseline self-rated health demonstrated weaker and less consistent associations with follow-up VAS scores. Household and social factors showed stronger and more consistent associations with VAS outcomes than infection status, including a significant association between comorbidities and lower follow-up VAS scores in the COVID-19 model. **Conclusions:** Among adults aged ?65 years with acute respiratory illness, respiratory virus test positivity was not associated with worse short-term HRQoL after adjustment for baseline health and social factors. Baseline health status and social context were more strongly associated with subsequent HRQoL and functional recovery than viral etiology.

**Figure:**
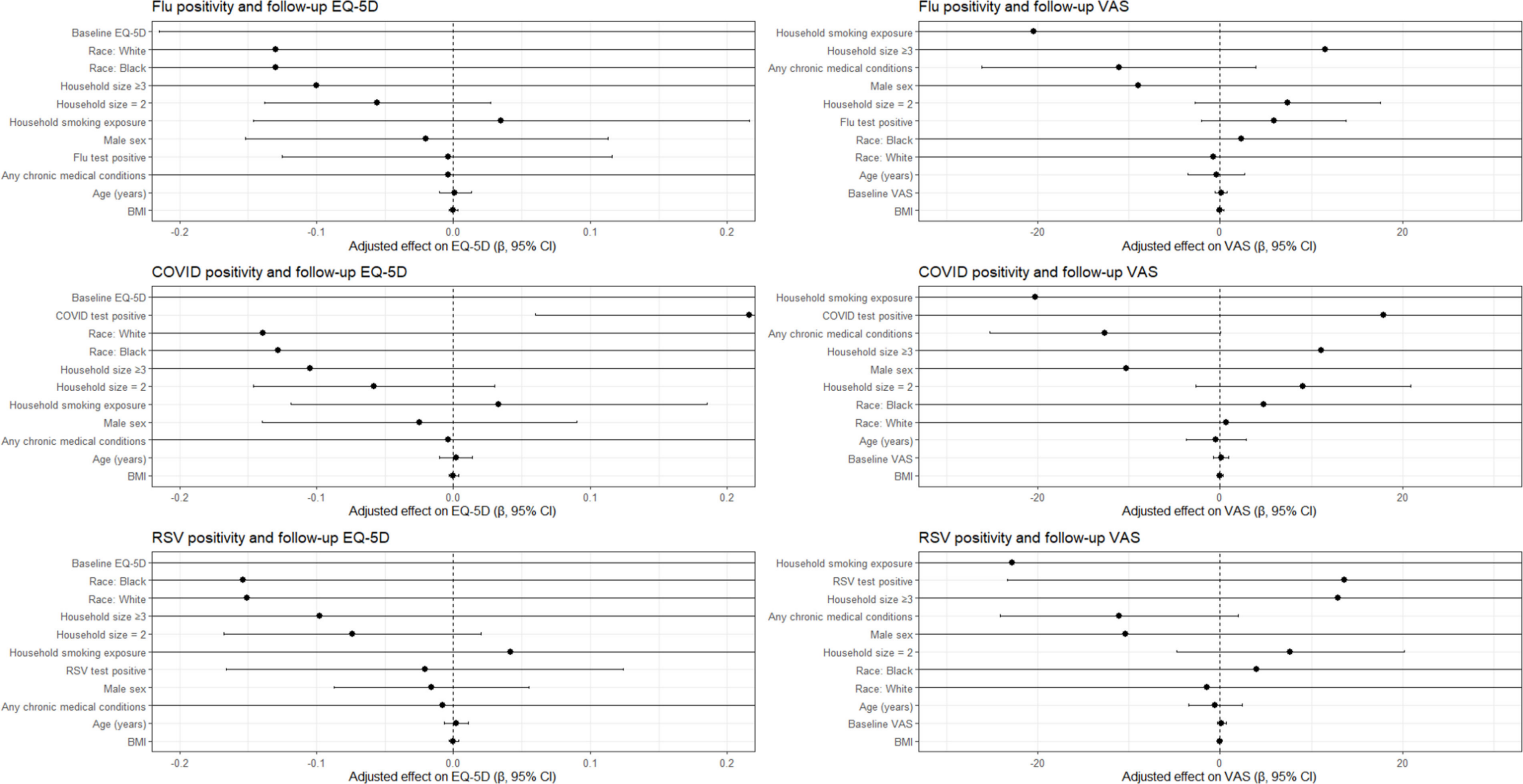

**Figure:**